# ChatGPT From the Perspective of an Academic Oral and Maxillofacial Radiologist

**DOI:** 10.7759/cureus.40053

**Published:** 2023-06-06

**Authors:** Sonam Khurana, Anusha Vaddi

**Affiliations:** 1 Oral Pathology, Radiology, and Medicine, New York University (NYU) College of Dentistry, New York, USA; 2 Oral and Maxillofacial Radiology, Virginia Commonwealth University School of Dentistry, Richmond, USA

**Keywords:** chatgpt, dental education, radiology report, maxillofacial radiology, scientific papers

## Abstract

Chat Generative Pre-Trained Transformer (ChatGPT) is an open artificial intelligence (AI)-powered chatbot with various clinical and academic dentistry applications, including oral and maxillofacial radiology (OMFR). The applications can be extended to generating documents such as oral radiology reports if appropriate prompts are given. There are various challenges associated with this task. Like other fields, ChatGPT can be incorporated to generate content and answer oral radiology-related multiple-choice questions. However, its performance is limited to answering image-based questions. ChatGPT can help in scientific writing but can not be designated as an author due to the lack of validity of the content. This editorial outlines the potential applications and limitations of the current version of ChatGPT in OMFR academic settings.

## Editorial

Chat Generative Pre-Trained Transformer, popularly known as ChatGPT, a novel open artificial intelligence (AI)-based chatbot tool, is controversial in academia. ChatGPT can generate extensive data and produce human-like responses because of its reinforcement feedback training. In simple words, ChatGPT makes it easier to communicate between computers and humans. ChatGPT is gaining interest in the scientific committee because it generates academic content and helps clinical decision-making [1].

ChatGPT has many applications in clinical dentistry and dental education. Its applications in dentistry can range from creating presentations to generating documents such as oral radiology reports. Oral and maxillofacial radiology (OMFR) is a specialty of dentistry that focuses on acquiring and interpreting images in the maxillofacial region that are used for diagnosis, treatment planning, and assessment of the prognosis. This paper provides an overview of ChatGPT through the lens of academic oral and maxillofacial radiologists. It focuses on the current scope of ChatGPT in dental education and its role in generating an oral radiology report.

ChatGPT in dental education

Dental faculty can use the algorithm to create presentations, develop grading rubrics, generate quizzes, and provide feedback on the student’s assignments. In addition, it can be used to draft emails and academic content to save time. Dental students can use the algorithm to make presentations and complete class assignments. The involvement of new technologies in a classroom depends on planning to implement them in the syllabi. Although there are many uses of ChatGPT, the piercing question among educators is, “Academia needs creative, competent teachers and students. Do we want them to be automated as robots?” Some faculty feel these tools might destroy the art and craft of writing if all students in a class start using ChatGPT for the same assignments, which can lead to plagiarism, copyright problems, and the authenticity of the information.

There are three possible solutions to avoid plagiarism and other potential problems due to ChatGPT.

Prevention

The faculty should clarify in their syllabi that using generative AI tools such as ChatGPT is prohibited. If students try to use it, it can lead to administrative actions against them. There are a variety of tools available to detect ChatGPT-generated material. However, these tools could be more reliable and have some limitations. The faculty should only partially depend on these tools in the decision-making process.

Restriction

Several tasks, such as oral presentation and adaptive learning methods, cannot be currently done with ChatGPT. The faculty can make it easier for the students to use ChatGPT to select topics for oral presentations and understand the content of adaptive learning assignments. The role of ChatGPT is limited in these scenarios and thus does not replace human efforts.

Inclusion

**Figure 1:**
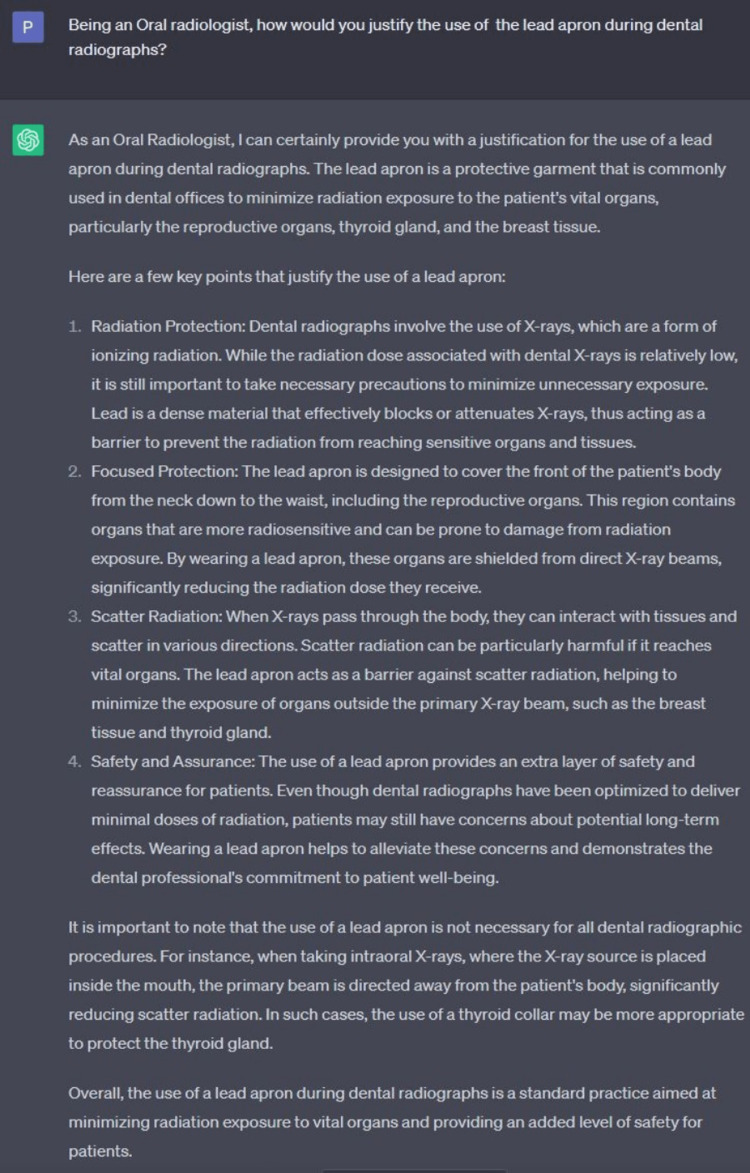
Screenshot from the current free version of ChatGPT demonstrating the ability to generate positive responses to a controversial topic ChatGPT: Chat Generative Pre-Trained Transformer

**Figure 2:**
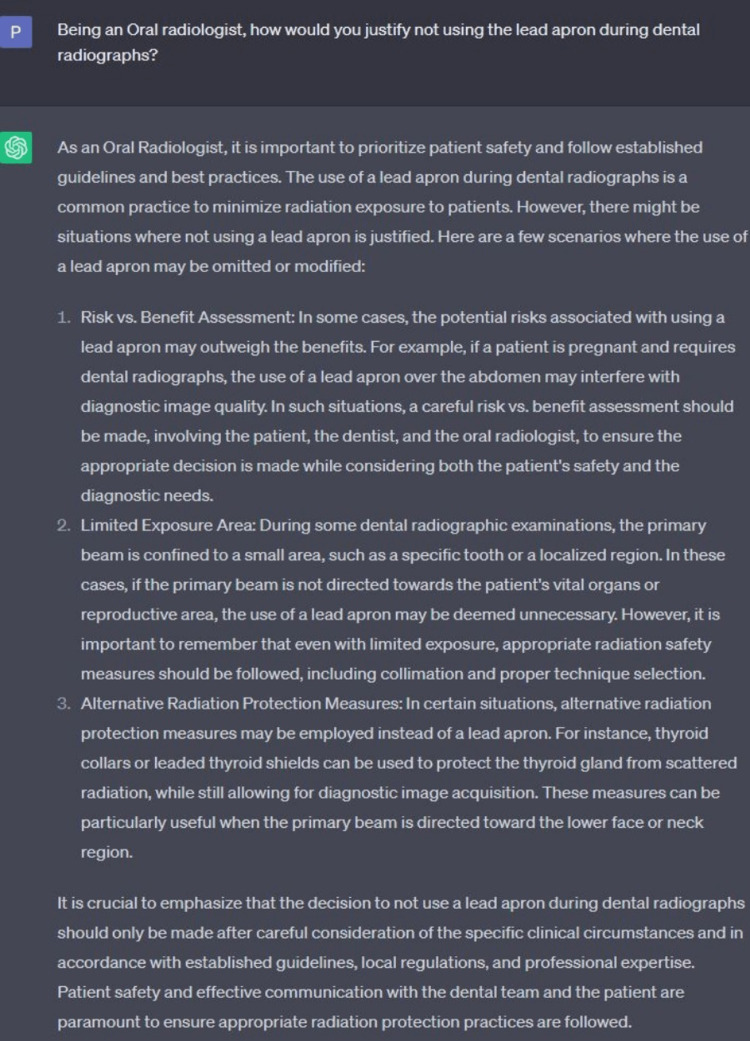
Screenshot from the current free version of ChatGPT demonstrating the ability to generate negative responses to a controversial topic ChatGPT: Chat Generative Pre-Trained Transformer

The faculty can use ChatGPT for controversial topics with pros and cons. ChatGPT can generate two types of essays, both in favor and against the issue, and students can conclude it in their own words. We have shown an example of a topic (the use of the lead apron in dental radiography) and generated two responses through ChatGPT, one in favor of using a lead apron and the other of not using it (Figure 1 and Figure 2). In this case, students must discuss ChatGPT-generated content versus their conclusion. It can be incorporated into group learning activities.

ChatGPT as a tool for answering examinations

Language models have a potential role in medical and dental education. ChatGPT performance was assessed for the United States Medical Licensing Examination (USMLE) Steps 1, 2, and 3 in a study. The examination consisted of open-ended questions with variable inputs and multiple-choice, single-answer questions with/without forced justification. ChatGPT performed lowest on the Step 1 examination, followed by Steps 2 and 3. The results are concordant with the human subjects and reflect the subjective comprehension and objectivity of the test takers. The concordance correlation coefficient was 90% for the correct answer choices [1].

**Figure 3:**
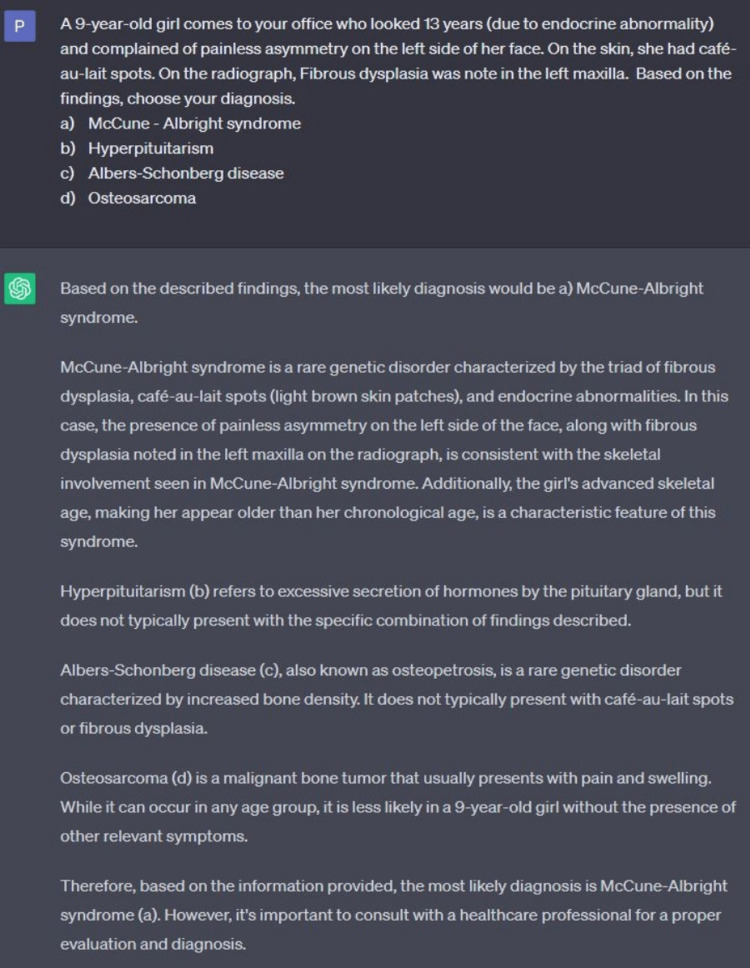
Screenshot from the current free version of ChatGPT demonstrating the ability to answer case-based question ChatGPT: Chat Generative Pre-Trained Transformer

**Figure 4:**
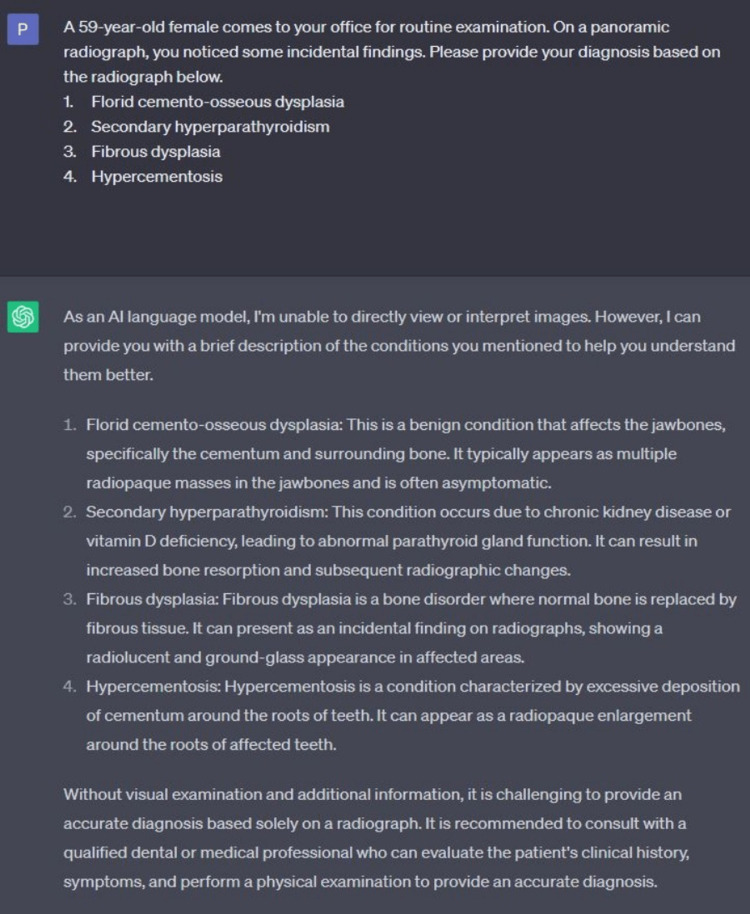
Screenshot from the current free version of ChatGPT demonstrating the ability to answer image-based question ChatGPT: Chat Generative Pre-Trained Transformer

We tested the ability of ChatGPT to answer oral radiology-based multiple-choice questions. ChatGPT performed well on questions based on the clinical information (Figure 3). The role of ChatGPT is limited when the information is provided in a pictorial format or through a radiograph (Figure 4). Our findings are in concordance with a recent study that assessed ChatGPT’s performance to assess a radiology board-style examination [2]. The role of ChatGPT can be tested to generate answers for Integrated National Board Dental Examination (INBDE) or specialty board examinations.

ChatGPT in maxillofacial radiology report writing

ChatGPT can generate a decent automated radiology report [3]. The radiologist must understand a few essential points before using ChatGPT for radiology report writing tasks.

**Figure 5:**
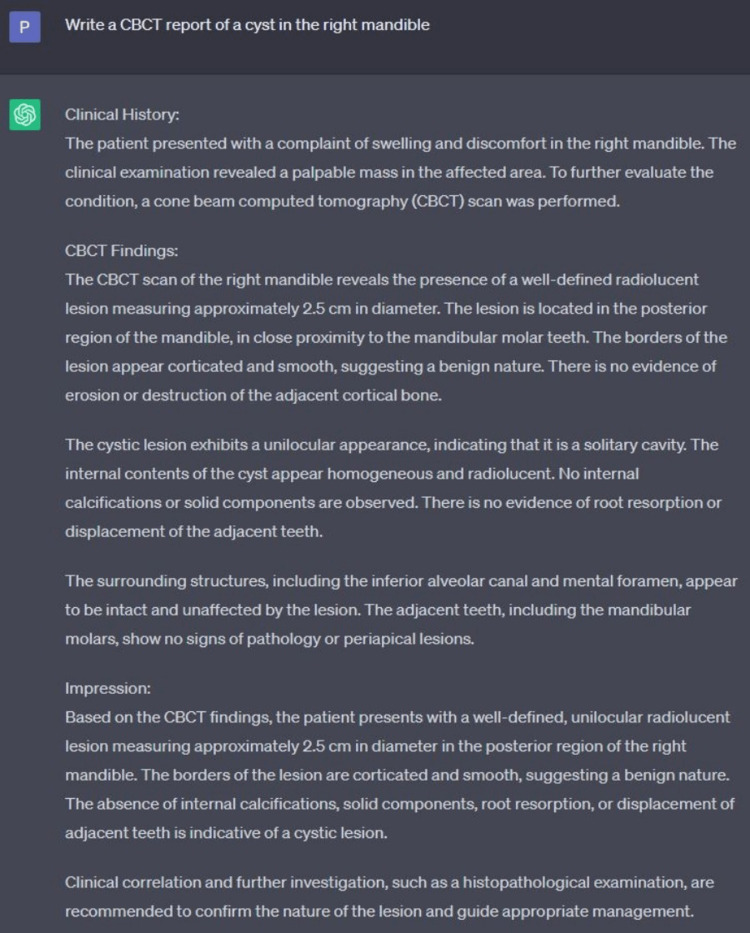
Screenshot from the current free version of ChatGPT showing the ability to generate preliminary radiology reports with limited prompts ChatGPT: Chat Generative Pre-Trained Transformer

**Figure 6:**
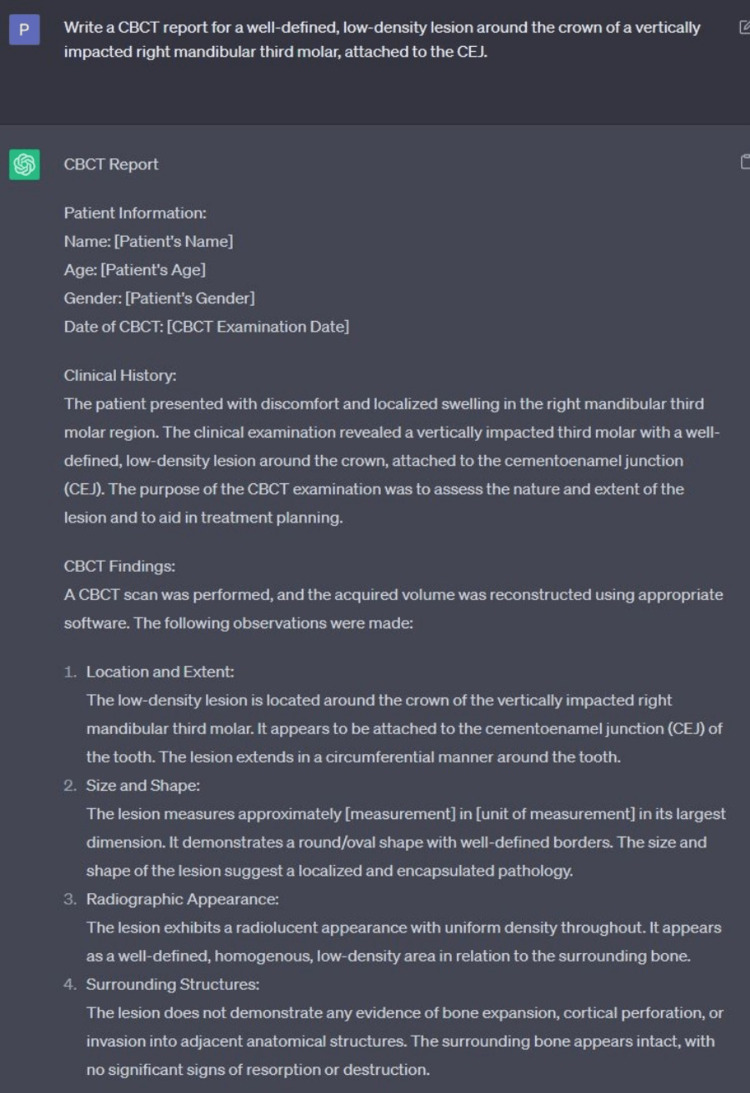
Screenshot from the current free version of ChatGPT showing the capability to generate preliminary radiology reports with appropriate prompts ChatGPT: Chat Generative Pre-Trained Transformer

**Figure 7:**
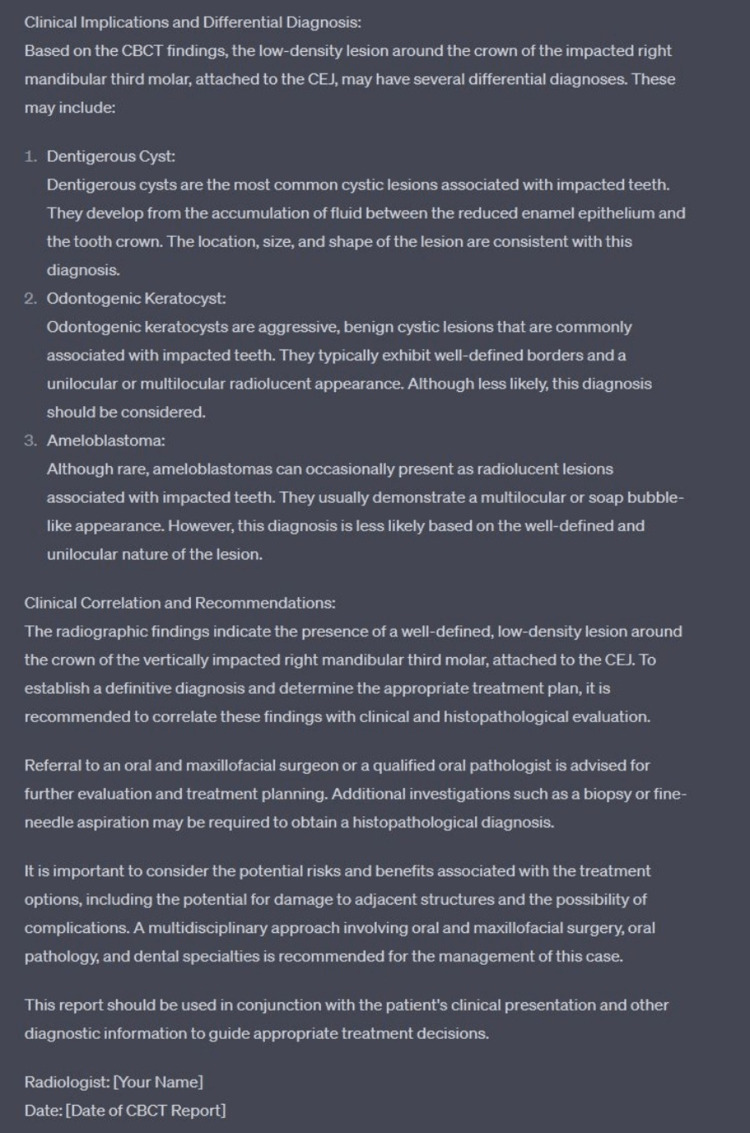
Continuation of preliminary radiology report from Figure 6 (screenshot from the current free version of ChatGPT showing the capability to generate preliminary radiology reports with appropriate prompts) ChatGPT: Chat Generative Pre-Trained Transformer

ChatGPT works on prompts provided by the radiologist. There are two examples given below. In the first example (Figure 5), the command was, “Write a cone beam computed tomography (CBCT) report of a cyst in the right mandible.” ChatGPT generated a preliminary report based on the prompt with many deficiencies without providing a differential diagnosis. In the second example (Figure 6 and Figure 7), the command was, “Write a CBCT report for a well-defined, low-density lesion around the crown of a vertically impacted right mandibular third molar, attached to the cementoenamel junction (CEJ).” The prompts in the second command were precise. The ChatGPT generated task-specific responses such as location, extent, size, shape, radiographic appearance, effect on surrounding structures, clinical implication, appropriate differential diagnosis, and recommendations.

As mentioned earlier, ChatGPT makes a reasonable response if commands are specific. However, relying on the report for clinical use is vicious. The radiologist has to remember that ChatGPT does not scroll through the scan to replace the radiologist’s work. In the second example, the ChatGPT made assumptions about the extent of the cyst and its effects on the surrounding structures. This is called “artificial hallucination in ChatGPT.” AI generates sentences to convince the reader, which can be misleading to the inexpert readers [1].

A radiologist has a medicolegal responsibility toward the radiology report. One must remember that ChatGPT is an AI-driven tool that does not replace the radiologist’s job and can not take responsibility for the content. The radiology report generated by the ChatGPT is adequate to be used as a draft. It can reduce the time to write a report in a busy practice. However, the radiologist should significantly edit the radiology report for clinical use and as a medicolegal document.

Role of ChatGPT in research and scientific writing

A recent systematic review evaluating the role of ChatGPT in healthcare education and research states that the tool was widely employed in scientific writing, analyzing large datasets followed by code generation, and creating rapid literature reviews. There is an ongoing debate regarding using ChatGPT as an author because it creates content that can be accurate or fictitious. Some authors called it an “AI-driven infodemic,” potentially threatening public health. ChatGPT is not yet qualified to be listed as an author. Nature Journal’s news team suggests recognizing the role of large language models (LLMs) under the acknowledgment section. The assistant director of Cold Spring Harbor Laboratory Press in New York discusses changing the usual belief that the author is not merely a document writer. The author’s responsibility includes integrity, validity, and legality of their work. Setting the code of ethics and best practices regarding using ChatGPT and other LLMs is the need of the hour. Nevertheless, these tools can generate summaries of published papers or highlight recommendations in an article. In addition, domain-specific models, such as “PubMedGPT,” trained exclusively on biomedical literature, will have a promising role in medical and dental education and research [4].

Conclusion

To summarize, the positive aspects of ChatGPT are ease of use and faster response rate, which saves the users’ time. The limitations of ChatGPT include its inherent inability to answer image-based questions and its lack of validation or authenticity of the content. To overcome some of these limitations, users should give appropriate prompts. In the case of radiology reports, providers should review the report thoroughly and make appropriate edits before signing off the report.

Although ChatGPT has a wide range of applications, its use in academia or for report writing is a long-term decision. This application can impose ethical and empirical questions in an educational environment. Since these tools are neoteric, some of the risks associated with usage are unknown yet. There is no short-term solution, and it requires the evolution of the models and our understanding of future adaptation. Future research should explore the benefits and shortcomings of incorporating LLMs into dental education and applying them to generate radiology reports. Currently, we live in an era of AI. Since this kind of generative AI tool has the potential to change the current teaching methods, academicians should be aware of the change and consider incorporating the tools if they have the potential to make a positive impact on student education.

If appropriate prompts are given, using ChatGPT to generate oral radiology reports saves the time of the provider. The most crucial factor is that the radiologist should review and edit the document before signing off. One of the future areas of interest would be to investigate the role of LLMs in decision-making processes, such as generating personalized treatment plans based on radiographic reports.
